# Diagnostic performance of TILs–US score and LPBC in biopsy specimens for predicting pathological complete response in patients with breast cancer

**DOI:** 10.1007/s10147-024-02634-9

**Published:** 2024-10-03

**Authors:** Hideo Shigematsu, Kayo Fukui, Akiko Kanou, Erika Yokoyama, Makiko Tanaka, Mutsumi Fujimoto, Kanako Suzuki, Haruka Ikejiri, Ai Amioka, Emiko Hiraoka, Shinsuke Sasada, Akiko Emi, Tetsuya Nakagiri, Koji Arihiro, Morihito Okada

**Affiliations:** 1 Department of Surgical Oncology, Research Institute for Radiation Biology and Medicine, Hiroshima University, 1-2-3-Kasumi, Minami-Ku, Hiroshima, 734-8551 Japan; 2 Division of Laboratory Medicine, Hiroshima University Hospital, Hiroshima, 734-8551 Japan; 3 Department of Breast Surgery, Hiroshima City North Medical Center Asa Citizens Hospital, Hiroshima, 731-0293 Japan; 4 Department of Anatomical Pathology, Hiroshima University Hospital, Hiroshima, 734-8551 Japan

**Keywords:** TILs, LPBC, Neoadjuvant chemotherapy, Pathological complete response, Breast cancer

## Abstract

**Background:**

Tumor-infiltrating lymphocytes–ultrasonography (TILs–US) score is used to predict lymphocyte-predominant breast cancer (LPBC) in surgical specimens. We aimed to compare diagnostic performance of TILs–US score for predicting pathological complete response (pCR) with that of LPBC in biopsy specimens.

**Methods:**

TILs ≥ 50% in biopsy specimens was defined as biopsy–LPBC, and TILs–US score ≥ 4 was categorized as TILs–US score-high. Basic nomogram for pCR was developed using stepwise logistic regression based on the smallest Akaike Information Criterion, and biopsy–LPBC and TILs–US score nomograms were developed by integrating biopsy–LPBC or TILs–US scores into a basic nomogram. The diagnostic performance of the nomograms for pCR was compared using area under the curve (AUC), categorical net reclassification improvement (NRI), and integrated discrimination improvement (IDI).

**Results:**

This retrospective study evaluated 118 patients with breast cancer, including 33 (28.0%) with biopsy–LPBC, 52 (44.1%) with TILs–US score-high, with 34 (28.8%) achieving pCR. The sensitivity, specificity, positive likelihood ratio, negative likelihood ratio, and AUC for predicting pCR were 0.53, 0.82, 2.96, 0.57, and 0.68, respectively, for biopsy–LPBC, and 0.76, 0.69, 2.47, 0.34, and 0.73, respectively, for TILs–US score. The biopsy–LPBC nomogram showed significant improvements in categorical NRI (*p* = 0.023) and IDI (*p* = 0.007) but not in AUC (*p* = 0.25), compared with the basic nomogram. The TILs–US nomogram exhibited significant improvements in AUC (*p* = 0.039), categorical NRI (*p* = 0.010), and IDI (*p* < 0.001).

**Conclusions:**

The TILs–US score may serve as a novel marker for prediction of pCR in patients with breast cancer. An external validation study is warranted to confirm our findings.

**Supplementary Information:**

The online version contains supplementary material available at 10.1007/s10147-024-02634-9.

## Introduction

Tumor-infiltrating lymphocytes (TILs) are lymphocytes that penetrate tumor tissues and serve as prognostic and predictive factors in various types of cancer [1–3]. Breast cancer with high TILs is referred to as lymphocyte–predominant breast cancer (LPBC), which has been reported as a favorable prognostic factor in early breast cancer [4, 5]. In breast cancer treated with neoadjuvant chemotherapy (NACT), the presence of LPBC in biopsy specimens is also associated with a high pathological complete response (pCR) rate [6, 7].

In patients with breast cancer treated with NACT, TILs are evaluated using needle biopsy specimens prior to NACT [8]. However, the evaluation of TILs in needle biopsy specimens has some limitations. First, needle biopsy of breast tumors is an invasive method, and repeating the needle biopsy is difficult in clinical practice [9]. Second, samples of needle biopsy represent a fraction of the tumor, potentially leading to an underestimation of TILs compared with TILs in surgical specimens [10, 11]. Finally, the assessment of TILs depends on pathologists’ subjective evaluation, which may lead to inter-observer discrepancies and lack of reproducibility [12].

The TILs–ultrasonography (US) score was developed as a non-invasive method to predict LPBC in surgical specimens [13]. The TILs–US score, calculated as the sum of scores for three ultrasonic tissue characteristics—shape, internal echo level, and posterior echoes—ranged from 0 to 7, with scores ≥ 4 categorized as TILs–US scores-high to predict LPBC in surgical specimens [13, 14]. A TILs–US score-high was shown as a significant factor for achieving pCR in patients treated with NACT [15]. Although the TILs–US score can be a novel and non-invasive method to predict LPBC, it remains unclear whether it offers diagnostic capabilities equivalent to those of needle biopsy in LPBC for predicting pCR.

This study aimed to examine whether the TILs–US score has equivalent diagnostic accuracy and clinical utilities compared with those of LPBC in needle biopsy specimens to predict pCR in breast cancer treated with NACT.

## Patients and methods

### Study design

This retrospective cohort study included patients treated with NACT for non-metastatic breast cancer at the Department of Breast Surgery, Hiroshima University Hospital, between May 2012 and November 2018. Eligibility criteria were the evaluation of TILs–US score and TILs in biopsy specimens prior to anthracycline- and taxane-based NACT followed by curative surgery. This research was carried out in accordance with the Declaration of Helsinki and the Clinical Research Law and was approved by the Ethics Committee of Hiroshima University (Approval number: E2018-1166). Given the retrospective observational nature of this study utilizing a hospital database, the requirement for informed consent was waived.

### Clinicopathological factors

Clinicopathological factors included age at diagnosis, histological type, clinical tumor, node, metastasis stage, nuclear grade, hormone receptor (HR) status, human epidermal growth factor receptor 2 (HER2) status, LPBC in biopsy specimens, TILs–US score, NACT regimen, and pCR. The cT and cN stages were determined according to the American Joint Committee on Cancer Staging Manual [16]. The histological type, nuclear grade, and the HR and HER2 status were assessed using needle biopsy specimens prior to NACT. HR positivity was defined as ≥ 1% staining for estrogen or progesterone receptors using immunohistochemistry, as per the American Society of Clinical Oncology/College of American Pathologists (ASCO/CAP) guidelines [17]. HER2 status was determined using immunohistochemistry (IHC) and/or in situ hybridization analysis; an IHC score of 3 + or HER2 gene amplification was considered HER2-positive, according to the ASCO/CAP guidelines [18].

Breast cancer subtypes were classified into three categories based on the expression of HR and HER2: HR-positive/HER2-negative, HER2-positive, and HR-negative/HER2-negative. The evaluation of TILs in needle biopsy specimens, conducted according to the 2014 recommendations of the International TILs Working Group, defined breast cancer with 50% or more TILs as biopsy–LPBC [8]. Biopsy–TILs were retrospectively assessed by the pathologist, KA and TN, using core needle biopsy specimens without clinical information. The TILs–US score was calculated based on breast ultrasound images prior to NACT [13]. Conventional ultrasound images were obtained using the HI VISION Ascendus and HI VISION Preirus systems (Hitachi, Chiyoda-ku, Tokyo, Japan), equipped with a linear-array transducer (5–18 MHz) for both longitudinal and transverse imaging. Patients were positioned supine with arms elevated during the image acquisition process**.** Representative images of lesions with the largest diameter were used to calculate the TILs–US score, as the sum of the points from three ultrasonic tissue characteristics: shape (round, oval, polygonal, irregular: 0 points; lobulated: 1 point; small lobulated: 2 points), internal echo level (high or equal: 0 points; low: 1 point; extremely low: 2 points), and posterior echoes (shadowing or attenuating: 0 points; unchanged: 1 point; accentuated: 2 points; very accentuated: 3 points). A TILs–US score ≥ 4 was defined as a TILs–US score-high in accordance with previous reports. Breast ultrasound examinations were conducted by medical laboratory technicians, KF and AK, and the TILs–US score was determined based on a consensus between clinicians and technologists. Anthracycline- and taxane-based regimens were administered as NACT. Trastuzumab was included in the NACT regimen for the treatment of patients with HER2-positive breast cancer. The pathological response was assessed using surgical specimens after NACT, defining pCR as the absence of residual invasive cancer and intraductal components in the breast and lymph nodes (ypT0 ypN0).

### Statistical analysis

The correlation between biopsy–LPBC and TILs–US score-high was assessed using the kappa (κ) statistic. The correlation between pCR and the clinicopathological factors was evaluated using logistic regression analysis. The odds ratios (OR) and 95% confidence interval (95% CI) were calculated using multivariate logistic regression analysis. The sensitivity, specificity, positive predictive value, negative predictive value, and area under the curve (AUC) of biopsy–TILs and TILs–US score-high for predicting pCR were evaluated. The predictor selection for the basic nomogram regarding pCR was conducted using backward variable selection based on the smallest Akaike information criterion (AIC), selecting predictors from clinical and pathological factors, excluding biopsy–LPBC and TILs–US score. The basic nomogram was then developed according to the logistic model using the selected predictors. As extended nomograms, biopsy–LPBC and TILs–US nomograms were developed by incorporating biopsy–LPBC or TILs–US score into these predictors. The discriminative abilities of the prediction nomograms for pCR were visualized using a receiver operating characteristic (ROC) curve, and the AUCs of the nomograms were compared using the DeLong test [19]. The calibration ability of the nomograms was adjusted using a calibration curve. The goodness of fit for the nomogram was checked using the Hosmer–Lemeshow test [20]. The clinical usefulness of the biopsy–LPBC nomogram and TILs–US nomogram compared with that of the basic nomogram was assessed using categorical net reclassification improvement (NRI), integrated discrimination improvement (IDI), and decision curve analysis (DCA) [21]. The interobserver agreement for biopsy–LPBC and TILs–US score-high was evaluated using the κ statistic. The strength of the κ agreement was categorized as follows: poor (κ < 0), slight (κ = 0.00–0.20), fair (κ = 0.21–0.40), moderate (κ = 0.41–0.60), substantial (κ = 0.61–0.80), and almost perfect (κ = 0.81–1.00) [22]. All statistical analyses were performed using the R software (version 4.2.3; https://www.Rproject.org/). Statistical significance was set at *p* < 0.05.

## Results

### Patient characteristics

During the study period, 215 patients with non-metastatic invasive breast cancer were treated with NACT. Biopsy–LPBC and TILs–US score were not assessed in 95 patients, including 90 who underwent needle biopsy at other institutions where TILs could not be evaluated on the samples and 5 who did not have breast ultrasonography prior to NACT due to completed imaging tests at other institutions. Two patients received non-anthracycline and taxane-based regimens for NACT. The remaining 118 patients were included in this study. Table 1 shows the clinicopathological characteristics of the patients. The participants’ median age was 51.5 years, and the predominant histological type was invasive ductal carcinoma (96.6%). The most frequent cT was T2, accounting for 66.1% of the cases, and more than half of the patients (55.9%) had cN + disease. The HR and HER2 positivity rates were 69.5% and 49.2%, respectively. HR-positive/HER2-negative, HER2-positive, and HR-negative/HER2-negative subtypes were observed in 36 (30.5%), 58 (49.2%), and 24 patients (20.3%), respectively. Nuclear Grade 3 tumors were the most prevalent (67.8% of the cases). Biopsy–LPBC was identified in 33 patients (28.0%), and a TILs–US score-high was observed in 52 patients (44.1%). The κ coefficient between biopsy–LPBC and TILs–US score-high was 0.30 (range: 0.14–0.47), suggesting a weak association between biopsy–LPBC and TILs–US score-high. pCR was achieved in 34 patients (28.8%).

**Table 1:** Clinicopathological characteristics

Factor	Total, 118 (100)
Age (years)	
Median 51.5 (interquartile range, 44–61)	
≤ 50	57 (48.3)
> 50	61 (51.7)
Histology	
Invasive ductal carcinoma	114 (96.6)
Special type*	4 (3.4)
Clinical T stage	
1	21 (17.8)
2	78 (66.1)
3	11 (9.3)
4	8 (6.8)
Clinical N stage	
0	52 (44.1)
1	44 (37.3)
2	14 (11.9)
3	8 (6.8)
HR	
Positive	82 (69.5)
Negative	36 (30.5)
HER2	
Positive	58 (49.2)
Negative	60 (50.8)
Subtype	
HR-positive, HER2-negative	**36 (30.5)**
HER2-positive	**58 (49.2)**
HR-negative, HER2-negative	**24 (20.3)**
Nuclear grade	
1	9 (7.6)
2	29 (24.6)
3	80 (67.8)
Biopsy–TILs (%)	
Median 20 (interquartile range, 5–52.5)	
LPBC (≥ 50%)	**33 (28.0)**
Non-LPBC (< 50%)	**85 (72.0)**
TILs–US score	
Median 3 (interquartile range, 2–5)	
TILs–US score-high (≥ 4)	**52 (44.1)**
TILs–US score-low (< 4)	**66 (55.9)**
Pathological complete response (ypT0N0)	
Yes	34 (28.8)
No	84 (71.2)

Data are expressed as *n* (%) unless otherwise specified

*HR* hormone receptor, *HER2* human epidermal growth factor receptor 2, *LPBC* lymphocyte-predominant breast cancer, *TILs–US* tumor-infiltrating lymphocytes–ultrasonography

^*^The special type includes two invasive lobular carcinomas, one mucinous carcinoma, one invasive micropapillary carcinoma, and one apocrine carcinoma

### Diagnostic accuracy of biopsy–LPBC and TILs–US score-high for the prediction of pCR

Table 2 presents the diagnostic accuracy of the biopsy–LPBC and TILs–US score-high for the prediction of pCR. The TILs–US score-high demonstrated numerically higher sensitivity (0.76 vs. 0.53) and a smaller negative likelihood ratio (0.34 vs. 0.57) than did biopsy–LPBC. In contrast, biopsy–LPBC showed numerically higher specificity (0.82 vs. 0.69) than did the TILs–US score-high. The accuracy and positive likelihood ratio for biopsy–LPBC and TILs–US score-high were 0.74 and 0.71, and 2.96 and 2.47, respectively. The AUC for biopsy–LPBC and TILs–US score-high were 0.68 and 0.73, respectively, with the DeLong test indicating no significant difference between these factors (*p* = 0.25). In each breast cancer subtype, the TILs–US score-high exhibited superior sensitivity and negative likelihood, compared with biopsy–LPBC (Supplemental Table 1). Conversely, equivalent values were noted for the accuracy and AUC between the TILs–US score-high and biopsy–LPBC.

**Table 2:** Diagnostic accuracy of the TILs–US score and biopsy–LPBC for predicting pathological complete response in whole population

Method	Threshold	Sensitivity	Specificity	Accuracy	Positive likelihood ratio	Negative likelihood ratio	Area under the curve
Biopsy–LPBC	LPBC vs. no LPBC	0.53 (0.35–0.70)	0.82 (0.72–0.90)	0.74 (0.65–0.81)	2.96 (1.70–5.18)	0.57 (0.40–0.83)	0.68 (0.58–0.77)
TILs–US score	high vs. low	0.76 (0.59–0.86)	0.69 (0.58–0.79)	0.71 (0.62–0.79)	2.47 (1.71–3.58)	0.34 (0.18–0.64)	0.73 (0.64–0.82)

95% confidence intervals in brackets

*LPBC* lymphocyte-predominant breast cancer, *TILs–US* tumor-infiltrating lymphocytes–ultrasonography

### Diagnostic performance of the nomograms for pCR

Table 3 shows the correlation between clinicopathological factors and pCR. In univariate analysis, cN0, NG3, HR-negative, biopsy–LPBC, and TILs–US score-high were significantly correlated with pCR. In the multivariate analysis, biopsy–LPBC and TILs–US score-high showed significant correlations with pCR, with ORs of 3.28 (95% CI, 1.19–9.24, *p* = 0.022) and 4.78 (95% CI, 1.81–13.6, *p* = 0.002), respectively. Other clinicopathological factors were not significantly correlated with pCR.

**Table 3:** Univariate and multivariate logistic regression analyses of factors associated with pathological complete response

Factor	Univariate analysis			Multivariate analysis		
	OR	95% CI	*p* value	OR	95% CI	*p* value
Age (years)						
> 55	1		0.27	1		0.35
≤ 55	1.59	0.70–3.62		1.63	0.59–4.70	
Clinical T stage						
2, 3, 4	1		0.3	1		0.97
1	1.68	0.63–4.52		1.02	0.31–3.65	
Clinical N stage						
1, 2, 3	1		0.014	1		0.24
0	2.76	1.21–6.28		1.81	0.68–4.90	
Nuclear grade						
1, 2	1		0.036	1		0.17
3	2.87	1.12–8.35		2.37	0.74–7.63	
Hormone receptor						
Positive	1		0.015	1		0.14
Negative	2.84	1.23–6.59		2.90	0.98–8.85	
Human epidermal growth factor receptor 2						
Negative	1		0.084	1		0.11
Positive	2.05	0.92–4.73		2.38	0.85–7.09	
Biopsy–LPBC						
LPBC	1		< 0.001	1		0.022
Non LPBC	5.18	2.18–12.7		3.28	1.19–9.24	
TILs–US score						
Low	1		< 0.001	1		0.002
High	7.25	3.00–19.2		4.78	1.81–13.6	

*OR* odds ratio, *CI* confidence interval, *LPBC* lymphocyte-predominant breast cancer, *TILs–US* tumor-infiltrating lymphocytes–ultrasonography

**Fig. 1:**
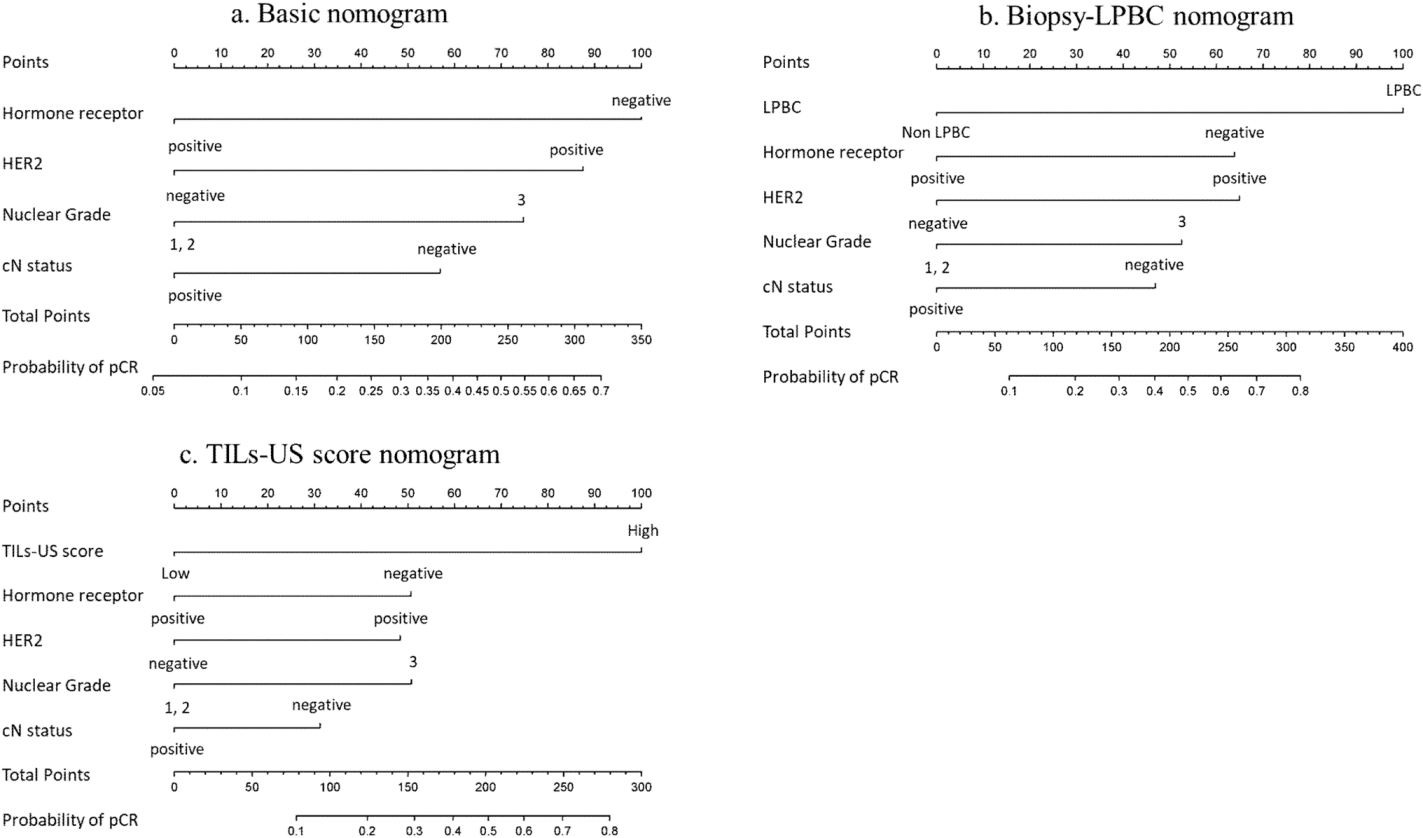
Basic, biopsy–LPBC, and TILs–US score nomograms to predict pathological complete response. *HER2* human epidermal growth factor 2, *pCR* pathological complete response, *LPBC* lymphocyte-predominant breast cancer, *TILs–US* tumor-infiltrating lymphocytes–ultrasonography, *CI* confidence interval, *AUC* area under the curve

**Fig. 2:**
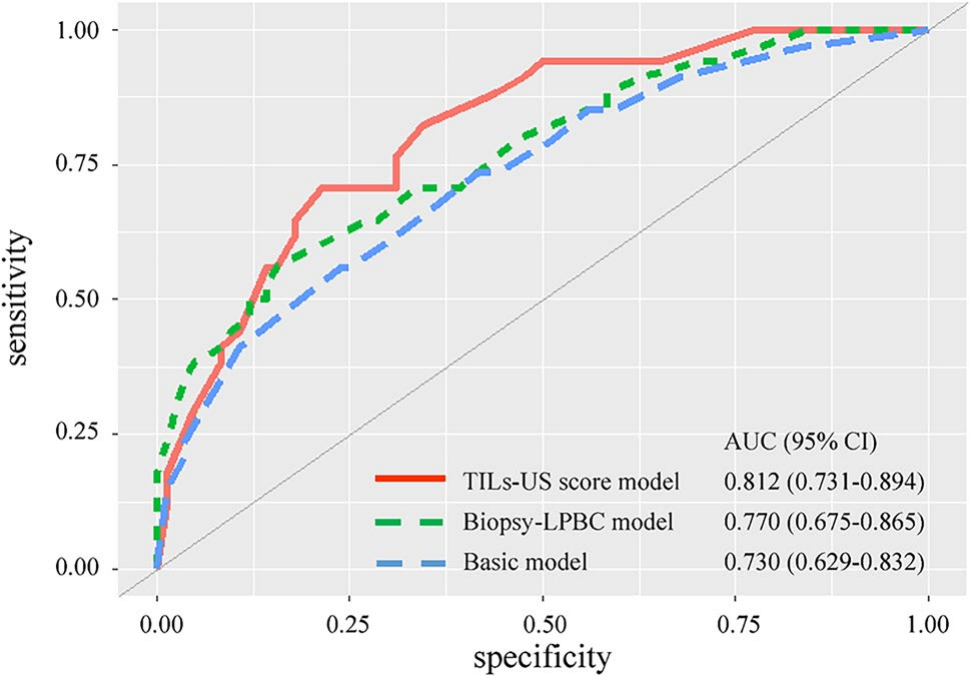
Receiver operation curves of prediction nomograms for a pathological complete response. The areas under curves are compared with the Delong test. *AUC* area under the curve, *LPBC* lymphocyte-predominant breast cancer, *TILs–US* tumor-infiltrating lymphocytes–ultrasonography

**Fig. 3:**
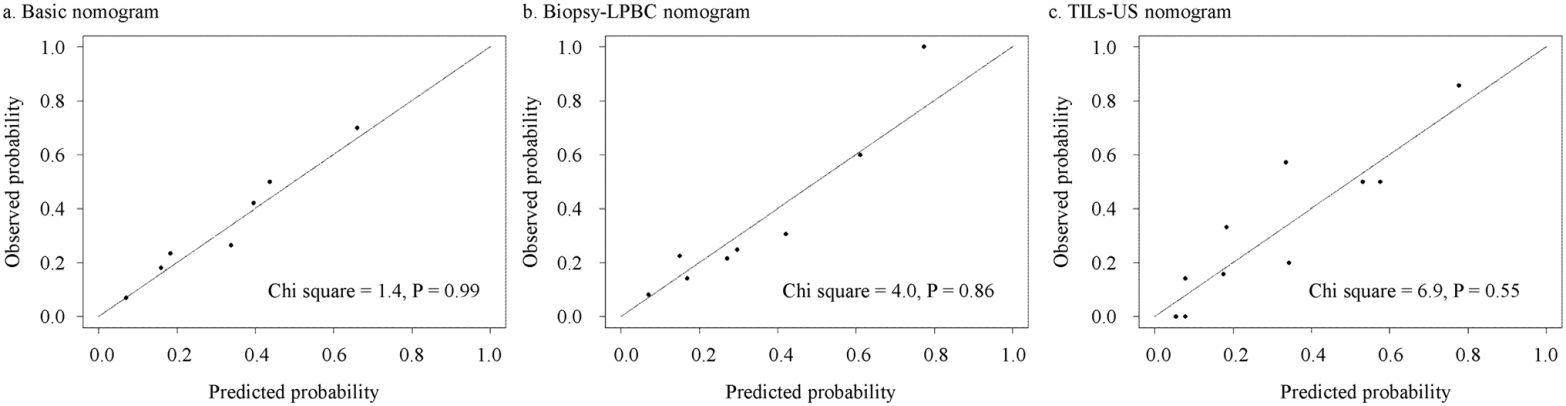
Calibration plots of prediction nomograms for a pathological complete response. *LPBC* lymphocyte-predominant breast cancer, *TILs–US* tumor-infiltrating lymphocytes–ultrasonography

**Fig. 4:**
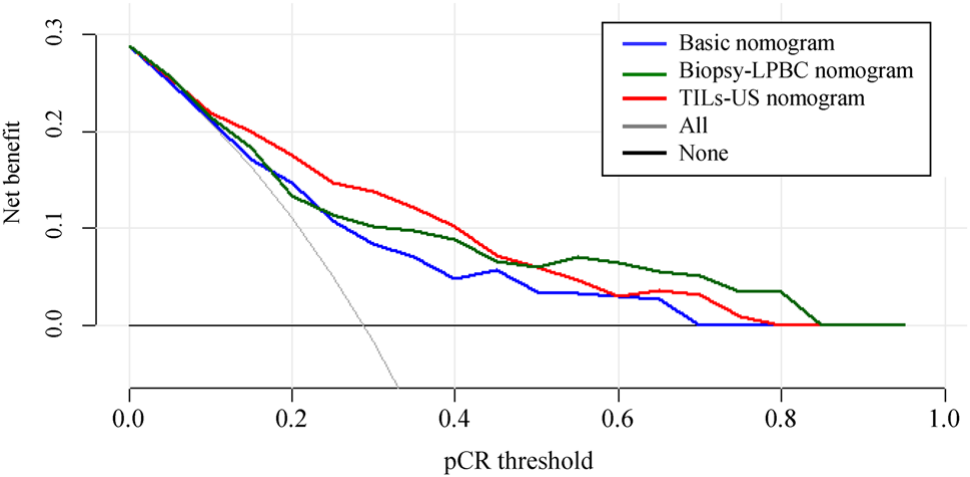
Decision curves of prediction nomograms for pCR. *LPBC* lymphocyte-predominant breast cancer, *TILs–US* tumor-infiltrating lymphocytes–ultrasonography, *pCR* pathological complete response

Age, cT, cN, NG, and HR and HER2 status were evaluated as predictor candidates of the basic nomogram for pCR. Backward variable selection was used to exclude predictors, and cN, HR and HER2 status, and NG were selected as predictors in the basic model, with the smallest AIC of 134.48. The basic nomogram was developed based on a logistic regression model for pCR using these predictors. The biopsy–LPBC nomogram, incorporating cN, HR and HER2 status, NG, and biopsy–LPBC, and the TILs–US score nomogram, incorporating cN, HR and HER2 status, NG, and the TILs–US score, were also developed. Figure 1 shows the basic, biopsy–LPBC and TILs–US score nomograms. Figure 2 shows the ROC curves for each nomogram. No significant difference in the AUC was observed between the biopsy–LPBC and basic nomograms (0.770 vs. 0.730, *p* = 0.25). However, the TILs–US nomogram showed a significant improvement in the AUC (0.812 vs. 0.730, *p* = 0.039), compared with the basic nomogram. In each breast cancer subtype, both the biopsy–LPBC and TILs–US score nomograms demonstrated improvements in the AUC, compared with the basic nomogram. Notably, in HER2-positive breast cancer, the increase in the AUC was particularly significant with the TILs–US score nomogram (Supplemental Fig. 1). Figure 3 shows the calibration plots of the prediction nomograms for a pCR. The calibration plots visually indicated a favorable fit between the observed and predicted probabilities of pCR for each nomogram. The Hosmer–Lemeshow Chi-square values for the basic, biopsy–LPBC, and TILs–US score nomograms were 1.4 (*p* = 0.99), 4.0 (*p* = 0.86), and 6.9 (*p* = 0.55), respectively. Figure 4 shows the decision curve of the nomograms. The DCA revealed that both the biopsy-LPBC and TILs–US score nomograms provided greater net benefit than did the basic nomogram across the threshold for pCR, ranging from 0.1 to 0.8. Table 3 compares the categorical net NRI and IDI for the biopsy–LPBC and TILs–US score nomograms with those of the basic nomogram. Both nomograms showed significant improvements in pCR prediction, with the biopsy–LPBC nomogram achieving a categorical NRI of 0.25 (*p* = 0.023) and IDI of 0.076 (*p* = 0.007), whereas the TILs–US score nomogram achieved a categorical NRI of 0.38 (*p* = 0.010) and IDI of 0.11 (*p* < 0.001), compared with the basic nomogram (Table 4).

**Table 4:** Comparison of the performance of nomograms for predicting pathological complete response

Model	Categorical net reclassification index		Integrated discrimination improvement	
	Value (95% CI)	*p* value	Value (95% CI)	*p* value
Basic nomogram	Reference		Reference	
Biopsy–LPBC nomogram	0.25 (0.034–0.46)	0.023	0.076 (0.021–0.13)	0.007
TILs–US nomogram	0.38 (0.090–0.66)	0.007	0.11 (0.051–0.17)	< 0.001

*LPBC* lymphocyte-predominant breast cancer, *TILs–US* tumor-infiltrating lymphocytes–ultrasonography, *CI* confidence interval

### Interobserver agreement for biopsy–LPBC and TILs–US score-high

The κ values for the assessment of biopsy–LPBC and TILs–US score-high were 0.81 (95% CI, 0.71–0.92) and 0.71 (95% CI, 0.59–0.84), respectively, indicating substantial agreement among the observers.

## Discussion

This retrospective study evaluated the diagnostic performance of biopsy–LPBC and the TILs–US score-high for predicting pCR in patients with breast cancer treated with NACT. The TILs–US score-high demonstrated diagnostic accuracy for pCR, which is equivalent to that of biopsy–LPBC, with numerically higher sensitivity and a smaller negative likelihood ratio. In the development of diagnostic prediction models for pCR, both biopsy–LPBC and the TILs–US score nomograms showed improvements in categorical NRI and IDI compared with basic nomogram; however, improvement in the AUC was observed only with the TILs–US score nomogram.

In studies of breast cancer treated with NACT, TILs in needle biopsy specimens have been used as a surrogate marker for TILs in surgical specimens [4, 7]. However, the levels of TILs in needle biopsy specimens have been reported to be lower than those in surgical specimens, resulting in an underestimation of LPBC [11, 12]. The sensitivity of biopsy–LPBC of surgical specimens has been reported to be approximately 0.5 [11, 14]. Furthermore, needle biopsy is an invasive method associated with risks of complications such as bleeding and pain [9]. To address these limitations, we developed the TILs–US score, a non-invasive method to predict LPBC in surgical specimens based on ultrasound findings [13]. In a previous study of breast cancer treated with surgery prior to systemic therapy, the TILs–US score-high demonstrated better discriminatory ability (AUC = 0.88) and higher sensitivity (0.74 vs. 0.51) for LPBC in surgical specimen than did biopsy–LPBC [14]. In this study, there was a weak agreement between TILs–US score-high and biopsy–LPBC (κ = 0.3). These findings suggest that the TILs–US score-high can be used as a non-invasive method to predict LPBC in surgical specimens independent of biopsy–LPBC, indicating that a TILs–US score-high may potentially serve as an alternative or complementary marker for biopsy–LPBC.

TILs levels were evaluated as a predictive marker for pCR in patients with breast cancer treated with NACT [4, 6, 23]. In a meta-analysis of randomized trials evaluating NACT, increased levels of TILs predicted a response to NACT across all breast cancer subtypes, with a high rate of pCR observed in the group with high TILs [4]. In our previous study focusing on HR-negative/HER2-negative and HER2-positive breast cancer, the TILs–US score-high was significantly correlated with pCR, showing a pCR rate of 75% in breast cancer with TILs–US score-high [15]. In this study involving all breast cancer subtypes, both biopsy–LPBC and TILs–US score-high were significantly correlated with pCR and demonstrated equivalent diagnostic accuracy. However, TILs–US score-high exhibited numerically higher sensitivity and a lower negative likelihood ratio compared with biopsy–LPBC. The increased sensitivity of the TILs–US score-high for surgical specimen LPBC may have contributed to the differences in diagnostic accuracy for pCR [14]. These findings suggest that the TILs–US score-high may serve as a predictive factor for the NACT of breast cancer, comparable to biopsy–TILs.

Diagnostic prediction models for pCR, which combine TILs in needle biopsy specimens with clinicopathological factors, have proven useful in patients with breast cancer treated with NACT [24–26]. These reports have developed prediction models using magnetic resonance imaging radiomic features, nutritional indices, or laboratory indicators along with TILs in needle biopsy specimens as predictors, demonstrating good discriminative power, calibration ability, and clinical utility for predicting pCR. This study developed a basic model based on conventional clinicopathological factors associated with pCR and examined the diagnostic improvement in an extended model that included either biopsy–LPBC or the TILs–US score-high. Based on the smallest AIC, cN, HR and HER2 status, and NG were selected as predictor candidates for the basic nomogram, all of which have been reported as factors associated with pCR [27, 28]. As extended nomograms, both biopsy–LPBC and the TILs–US score nomograms showed improvements in categorical NRI and IDI, compared with basic model, demonstrating their clinical utility to predict pCR. TILs–US score-high also improved the discriminative ability with improved AUC. These findings suggested that TILs–US score-high could serve as a new factor in the development of prediction models for pCR. In addition, as TILs–US score-high and biopsy–LPBC exhibit a weak correlation, a predictive model that incorporates both is expected to improve the diagnostic performance in predicting pCR. Further studies are warranted to evaluate predictive models for pCR that incorporate the TILs–US score-high and biopsy–LPBC.

One of the primary objectives in predicting pCR is to identify candidates for non-excisional therapies, in which a false-negative rate below 5% is considered acceptable in eligibility decisions [29, 30]. Although several studies have assessed the diagnostic capability of needle biopsies on the tumor bed post-NACT for predicting pCR, needle biopsy alone was unable to achieve the required diagnostic accuracy [29–33]. In ongoing clinical trials of non-excisional therapy, eligibility decisions are made by integrating needle biopsy pCR with imaging findings and subtype classification [34, 35]. This study highlights the added value of the TILs–US score to clinicopathological factors in prediction of pCR, suggesting that a predictive model combining the TILs–US score with biopsy–LPBC may improve the ability to predict pCR. Prospective studies are warranted to confirm the clinical implication of the TILs–US score in selecting candidates for non-excisional therapy.

This study has some limitations. First, owing to the single-center nature of this study, generalizability of the results may be limited. Furthermore, being a retrospective cohort study, potential risks of missing medical information and selection bias can exist. Indeed, nearly half of the breast cancer cases treated with NACT were excluded due to eligibility issues, potentially resulting in selection bias. Second, the limited number of cases in this study precluded external validation. Although internal validation was conducted, the possibility of overfitting cannot be eliminated. To confirm the results of this study, external validation on cohorts with consideration of adequate statistical power is warranted.

The findings of this study indicated that the TILs–US score-high could demonstrate diagnostic ability and clinical utility comparable to that of biopsy–LPBC for predicting pCR in patients with breast cancer treated with NACT. The TILs–US score is a non-invasive parameter to predict LPBC in surgical specimen and TILs–US score-high could be a novel factor to predict pCR. However, given the limitations of a single-center retrospective study without external validation, further validation studies including a multi-center prospective cohort are essential to confirm our findings.

## Supplementary information

Below is the link to the electronic supplementary material.

Supplementary file1 Supplemental Fig. 1 Receiver operating characteristic curves of prediction nomograms for a pathological complete response in each breast cancer subtype. The areas under curves are compared using the DeLong test in the (a) HR-positive/HER2-negative, (b) HER2-positive and (c) HR-negative/HER2-negative subtypes. HR, hormone receptor; HER2, human epidermal growth factor receptor 2; AUC, area under the curve; LPBC, lymphocyte-predominant breast cancer; TILs-US, tumor-infiltrating lymphocytes-ultrasonography (TIFF 2037 KB)

Supplementary file2 (DOCX 17 KB)

## Data Availability

The data sets used in this study are available from the corresponding author upon request.
