# Supplementary figures and images for: Diagnostic performance of TILs–US score and LPBC in biopsy specimens for predicting pathological complete response in patients with breast cancer
Source: Int J Clin Oncol. 2024 Oct 3;29(12):1860–9. doi: 10.1007/s10147-024-02634-9 (PMC11588827; doi:10.1007/s10147-024-02634-9)

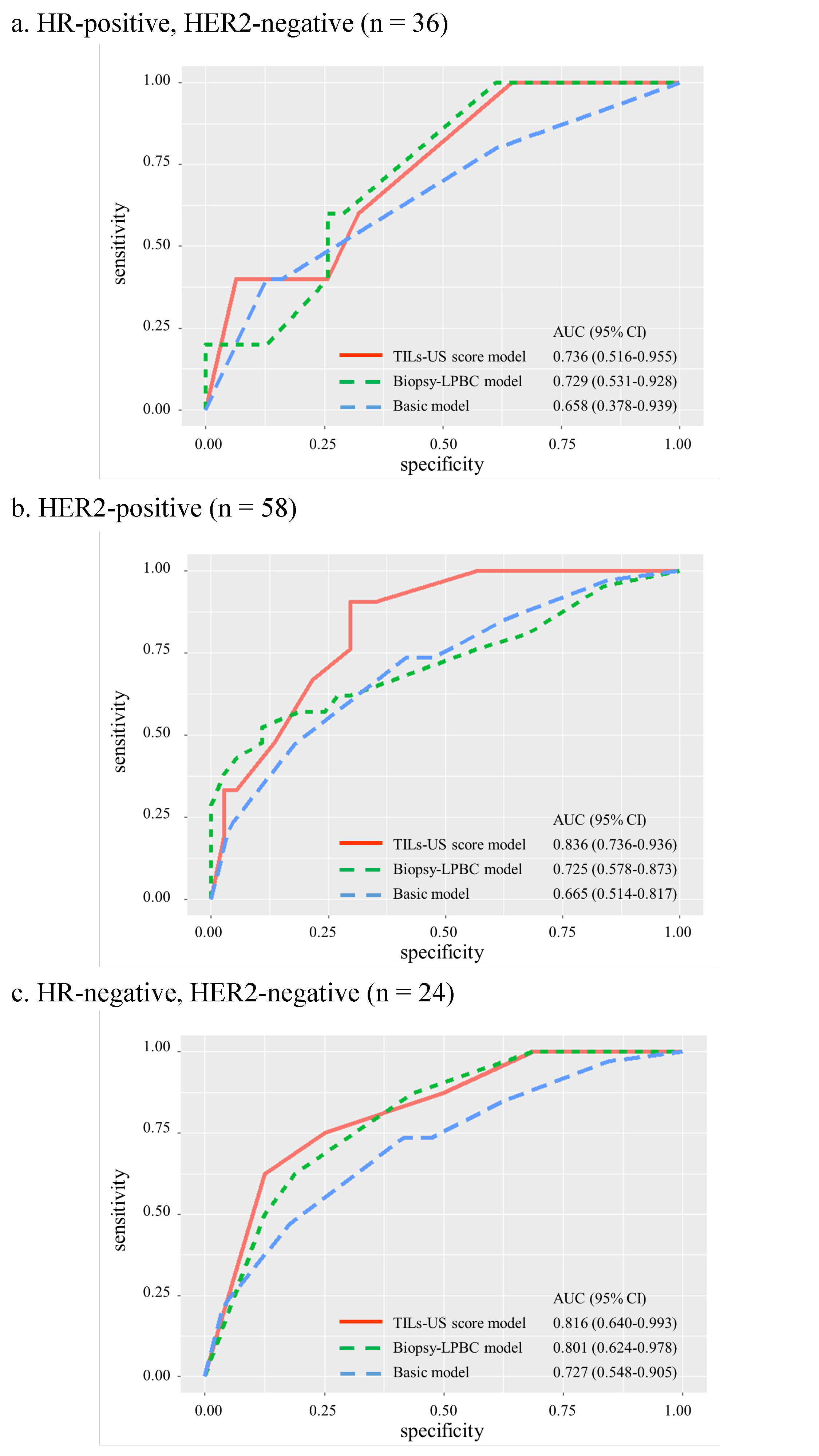

Supplement: Supplementary file 1 — Supplementary file1 Supplemental Fig. 1 Receiver operating characteristic curves of prediction nomograms for a pathological complete response in each breast cancer subtype. The areas under curves are compared using the DeLong test in the (a) HR-positive/HER2-negative, (b) HER2-positive and (c) HR-negative/HER2-negative subtypes. HR, hormone receptor; HER2, human epidermal growth factor receptor 2; AUC, area under the curve; LPBC, lymphocyte-predominant breast cancer; TILs-US, tumor-infiltrating lymphocytes-ultrasonography (TIFF 2037 KB) [file 10147_2024_2634_MOESM1_ESM.tiff]
