# Supplementary material for: Diagnostic performance of TILs–US score and LPBC in biopsy specimens for predicting pathological complete response in patients with breast cancer
Source: Int J Clin Oncol. 2024 Oct 3;29(12):1860–9. doi: 10.1007/s10147-024-02634-9 (PMC11588827; doi:10.1007/s10147-024-02634-9)
Supplement: Supplementary file 2 — Supplementary file2 (DOCX 17 KB) [file 10147_2024_2634_MOESM2_ESM.docx]

**Supplemental Table 1. The diagnostic accuracy of the TILs-US score and biopsy-LPBC for predicting pathological complete response in HR-positive, HER2-negative (a), HER2-positive (b) and HR-negative, HER2-negative breast cancer (c).**

a. HR-positive, HER2-negative (n = 36)

| Method | Threshold | Sensitivity | Specificity | Accuracy | Positive likelihood ratio | Negative likelihood ratio | Area under curve |
| --- | --- | --- | --- | --- | --- | --- | --- |
| Biopsy-LPBC | LPBC vs. no LPBC | 0.40  (0.05―0.85) | 0.87  (0.70―0.96) | 0.81  (0.64―0.92) | 3.10  (0.76―12.7) | 0.69  (0.33―1.43) | 0.64  (0.39―0.88) |
| TILs-US score | high vs. low | 0.60  (0.15―0.95) | 0.74  (0.55―0.88) | 0.72  (0.55―0.86) | 2.33  (0.92―5.90) | 0.54  (0.18―1.61) | 0.67  (0.55―0.86) |

b. HER2-positive (n = 58)

| Method | Threshold | Sensitivity | Specificity | Accuracy | Positive likelihood ratio | Negative likelihood ratio | Area under curve |
| --- | --- | --- | --- | --- | --- | --- | --- |
| Biopsy-LPBC | LPBC vs. no LPBC | 0.53  (0.35―0.70) | 0.82  (0.72―0.90) | 0.74  (0.65―0.81) | 2.96  (1.70―5.18) | 0.57  (0.40―0.83) | 0.67  (0.54―0.79) |
| TILs-US score | high vs. low | 0.86  (0.64―0.97) | 0.70  (0.53―0.84) | 0.76  (0.63―0.86) | 2.88  (1.71―4.88) | 0.20  (0.07―0.59) | 0.78  (0.67―0.89) |

c. HR-negative, HER2-negative (n = 24)

| Method | Threshold | Sensitivity | Specificity | Accuracy | Positive likelihood ratio | Negative likelihood ratio | Area under curve |
| --- | --- | --- | --- | --- | --- | --- | --- |
| Biopsy-LPBC | LPBC vs. no LPBC | 0.62  (0.24―0.91) | 0.75  (0.48―0.93) | 0.71  (0.49―0.87) | 2.50  (0.92―6.82) | 0.50  (0.20―1.28) | 0.69  (0.48―0.90) |
| TILs-US score | high vs. low | 0.75  (0.35―0.97) | 0.62  (0.35―0.85) | 0.67  (0.45―0.84) | 2.00  (0.95―4.23) | 0.40  (0.11―1.41) | 0.69  (0.49―0.89) |

95% confidence intervals in brackets.

HR, hormone receptor; HER2, human epidermal growth factor receptor 2
